# Biomechanical and structural responses of the aorta to intermittent hypobaric hypoxia in a rat model

**DOI:** 10.1038/s41598-022-07616-3

**Published:** 2022-03-08

**Authors:** Andrés Utrera, Álvaro Navarrete, Alejandro González-Candia, Claudio García-Herrera, Emilio A. Herrera

**Affiliations:** 1 Departamento de Ingeniería Mecánica, Universidad de Santiago de Chile, Santiago, Chile; 2 Instituto de Ciencias de La Salud, Universidad de O’Higgins, Rancagua, Chile; 3 Programa de Fisiopatología, ICBM, Facultad de Medicina, Universidad de Chile, Santiago, Chile; 4 International Center for Andean Studies (INCAS), Universidad de Chile, Santiago, Chile

**Keywords:** Biophysics, Biomedical engineering, Experimental models of disease

## Abstract

High altitude hypoxia is a condition experienced by diverse populations worldwide. In addition, several jobs require working shifts where workers are exposed to repetitive cycles of hypobaric hypoxia and normobaric normoxia. Currently, few is known about the biomechanical cardiovascular responses of this condition. In the present study, we investigate the cycle-dependent biomechanical effects of intermittent hypobaric hypoxia (IHH) on the thoracic aorta artery, in terms of both structure and function. To determine the vascular effects of IHH, functional, mechanical and histological approaches were carried out in the thoracic aorta artery, using uniaxial, pre-stretch, ring opening, myography, and histological tests. Three groups of rats were established: control (normobaric normoxia, NN), 4-cycles of intermittent hypoxia (short-term intermittent hypobaric hypoxia, STH), and 10-cycles of intermittent hypoxia (long-term intermittent hypobaric hypoxia, LTH). The pre-stretch and ring opening tests, aimed at quantifying residual strains of the tissues in longitudinal and circumferential directions, showed that the hypoxia condition leads to an increase in the longitudinal stretch and a marked decrease of the circumferential residual strain. The uniaxial mechanical tests were used to determine the elastic properties of the tissues, showing that a general stiffening process occurs during the early stages of the IH (STH group), specially leading to a significative increase in the high strain elastic modulus ($E_{2}$) and an increasing trend of low strain elastic modulus ($E_{1}$). In contrast, the LTH group showed a more control-like mechanical behavior. Myography test, used to assess the vasoactive function, revealed that IH induces a high sensitivity to vasoconstrictor agents as a function of hypoxic cycles. In addition, the aorta showed an increased muscle-dependent vasorelaxation on the LTH group. Histological tests, used to quantify the elastic fiber, nuclei, and geometrical properties, showed that the STH group presents a state of vascular fibrosis, with a significant increase in elastin content, and a tendency towards an increase in collagen fibers. In addition, advanced stages of IH (LTH), showed a vascular remodeling effect with a significant increase of internal and external diameters. Considering all the multidimensional vascular effects, we propose the existence of a long-term passive adaptation mechanism and vascular dysfunction as cycle-dependent effects of intermittent exposures to hypobaric hypoxia.

## Introduction

Hypoxic conditions can be induced by several conditions such as stroke, emphysema, sleep-apnea, and living at extreme altitude^1^. Hypobaric hypoxia (HH), particularly referred to high altitude hypoxia, is related to a decrease in the atmospheric or barometric pressure (BP) relative to sea-level conditions, inducing several multisystemic health issues and potential risks^2^. The effect of HH by high altitude conditions is physiologically and clinically relevant over 2500 m.a.s.l, due to a drop in arterial oxygen pressure (PaO_2_) and saturation (SaO_2_), whose factors, in combination with the system hemodynamical and respiratory responses, conditions the physiological alterations regarding HH^3,4^. According to previous studies, exposure to long-term HH may lead to *high-altitude illness*, such as Acute Mountain Sickness (AMS), High Altitude Pulmonary Edema (HAPE) and High Altitude Cerebral Edema (HACE)^5,6^, among other pathophysiological conditions. It is estimated that more than 170 million people live at more than 2500 m.a.s.l around the world; of these, nearly 40 million in South America (Andean mountains), where the highest population density is found above 3500 m.a.s.l.^2,7^.

A specific model related to hypoxic condition corresponds to intermittent hypoxia (IH), considered as a “repetitive hypoxia interspersed with episodes of normoxia”^8^. One specific IH model corresponds to chronic intermittent hypoxia (CIH), characteristic to individuals who work in shift systems at high altitude, alternating high altitude working periods with resting periods at sea level. This condition can be observed in the Chilean mining activities on the Andean Mountains, especially in copper extraction, since it is one of the largest copper producing countries in the world, with approximately 16 mining operations above 3000 m.a.s.l. By 2019, Chile held a total working force of 248,803 miners, including men and women, where 34.3% of the workers had 7 × 7 shifts (seven days of work, followed by seven days of rest at sea level) and a 30.3% of them had 4 × 4 shifts, in a schedule that last several years. Other examples of CIH condition are observed in sports activities, astronomical observatories, military, customs, and border control in countries with high-altitude areas along the world^9^.

The physiological response of individuals subjected to high altitude intermittent exposure to hypoxia are still under study and has gained attention in recent years. In particular, animal models have described several mechanisms involved in hypobaric hypoxia-induced pulmonary hypertension^10,11^, specifically in rats^12,13^. Adaptative or detrimental effects of intermittent hypoxia can be assessed by intensity, frequency and duration of the hypobaric cycles^2^. In this sense, animal models have shown that initial intermittent hypobaric hypoxia (IHH) states are beneficial, inducing improved cardiovascular function and protection^14^. For instance, short exposures to IHH induced protection in cells, tissues, and organs evidenced when exposed to severe hypoxia and ischemia^15^. However, when hypoxia extends in time, chronic cellular response activation induces an acclimatization response that may induce maladaptive changes, with an increased cardiovascular risk^2^. Farías et al.^16^, describe an acclimatization process that causes a multisystemic adjustment over a given time, in which pulmonary vascular tone, cardiovascular response, exercise response, and oxidative stress are affected by the IHH. In addition, Zhang et al.^17^, suggest an enhanced arterial oxygen delivery, based on cumulative effects of repeated IH exposures, which includes an increased heart rate and normalization of the SaO_2_. Moreover, Thomas et al.^18^ examined vasoactive agents in arteries of rats exposed to IH, observing alterations in pulmonary vascular function, an elevated right ventricular systolic pressure, and a presumably pulmonary hypertension, but no changes in aortic function. Finally, Herrera et al.^14^, determined the combined effect of Omega 3 supplementation and IH on cardiac function, suggesting that IH and Omega 3, in an independent manner, generate an antioxidant and anti-inflammatory mechanism that promotes cardioprotection. Remodeling and mechanical properties alterations of biological tissues, when faced to environmental changes, are related with changes in the different microstructural components. For instance, in arteries, these changes are associated to collagen and elastin deposition, and smooth muscle cells (SMC) densities, with detrimental effects associated with vascular impairment and cardiovascular diseases^4^.

From a biomechanical point of view, soft tissue mechanical characterization has been useful in determining disease progression, potential risk of pathologies, a guide for surgical interventions, the development of tissue-engineered arterial grafts, among other areas of benefits^19–21^. Several studies have evaluated the biomechanics of soft tissues exposed to chronic hypoxia. Drexler et al.^22^ determine the effects chronic hypoxia on the mechanical properties of main pulmonary artery and the right and left branches (extrapulmonary arteries) through the inflation test, concluding that chronic hypoxic pulmonary hypertension is associated with the stiffening of all extrapulmonary arteries. In addition, Kobs et al.^23^ studied the remodeling effect in pulmonary arteries of mice subjected to hypoxia-induced hypertension through a pressurization test. The authors concluded that an enhanced elastin and collagen deposits, and thicknening of the arterial wall was associated with an increased stiffness due to hypoxia. On a different study, Navarrete et al.^4^ determined residual strain levels, through the ring opening test and pre-stretching test, in sheep exposed to chronic hypoxia during gestation and treated with experimental vasodilators. The authors studied the effects on neonates born from hypoxic ewes, establishing a relationship between medical treatments and beneficial mechanical response of the arterial wall. In a similar way, Rivera et al.^24^ evaluated the passive mechanical responses of arteries using the uniaxial tensile test was after an experimental treatment with melatonin in chronic hypoxic neonates. Although they did not found any differences in the mechanical properties, histological analyses showed that melatonin decreases cell proliferation, showing an antiremodeling capacity. However, the IHH effects on the mechanical behavior of cardiovascular tissues are still widely unknown. To the best of our knowledge, there is only one report of biomechanical cardiovascular effects of IHH^15^, where authors describe vascular function and biomechanical properties in a rat model subjected to 4 cycles of hypobaric intermittent hypoxia, using a wire myograph. Specifically, femoral vessels exhibited less stiffening and improved vasodilator capacity, suggesting that the initial stages of IH are beneficial for cardiovascular function. To cover a broader spectrum in biomechanical analysis, the current research explores the mechanical responses and properties of the thoracic aorta through residual strain and uniaxial tensile test, correlating these results with ex vivo function by wire myography, and with the microstructure of the arterial wall through histological analysis. Due to the lack of cardiovascular biomechanical studies under IHH condition, this research offers a valuable contribution of an integral vascular response to high-altitude exposure.

## Materials and methods

### Animals

**Figure 1:**
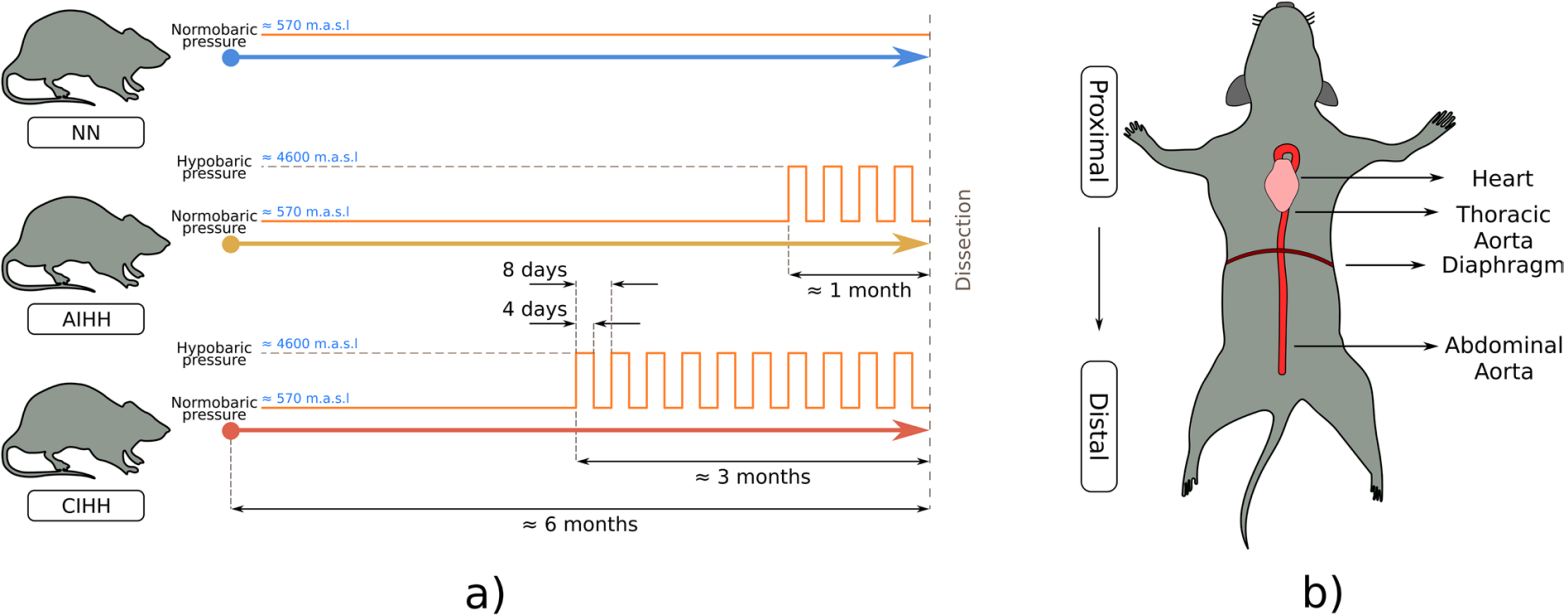
Experimental exposure to hypoxic cycles (**a**) and aorta topological overview (**b**).

This study was approved by the Institutional Animal Care and Use Committee (IACUC) of the University of Chile (Protocol CBA 0865 FMUCH). All the procedures involving animals were carried out in accordance with the Guide for the Care and Use of Laboratory Animals published by the US National Institutes of Health (NIH Publication No. 85-23, revised 1996) and the ARRIVE Guidelines^25^. Eighteen adult male Wistar rats were housed in standard cages in a temperature and light controlled room (22–24 °C; 12 h of light, 12 h of dark). Rats were randomly assigned to the following groups: normobaric normoxia (NN) (n = 6 rats), short-term intermittent hypobaric hypoxia (STH) (n = 6 rats) for 4 cycles and long-term intermittent hypobaric hypoxia (LTH) (n = 6 rats) for 10 cycles (Fig. 1a). Each cycle consists of 4 days of hypobaric hypoxia followed by 4 days of normobaric normoxia. The desired environmental pressure of the hypobaric chamber was achieved by pressure changes simulating altitude increases of 150 m per minute, until reaching 4600 m.a.s.l. (~ 428 torr). All animals received the same amount of daily food and drinking water (ad libitum pellets and water). At the end of the experimental protocol, 6 months-old animals were euthanized, with an anesthetic overdose (Sodium Thiopentone 150 mg kg^−1^ IP) and the aorta artery was extracted to perform biomechanical, functional and histological tests (Fig. 1b).

### Experimental procedures

All ex vivo tests (biomechanical & functional) were carried out in the aorta within 1 h after euthanasia. All mechanical tests described below were performed maintaining the specimens submerged in a Ca-free Krebs buffer at 38 °C to replicate physiological conditions^4,24^.

#### Residual strain tests

Two different tests were performed to obtain residual stress levels in longitudinal and circumferential directions: axial pre-stretch test and ring opening test, respectively.

**Figure 2:**
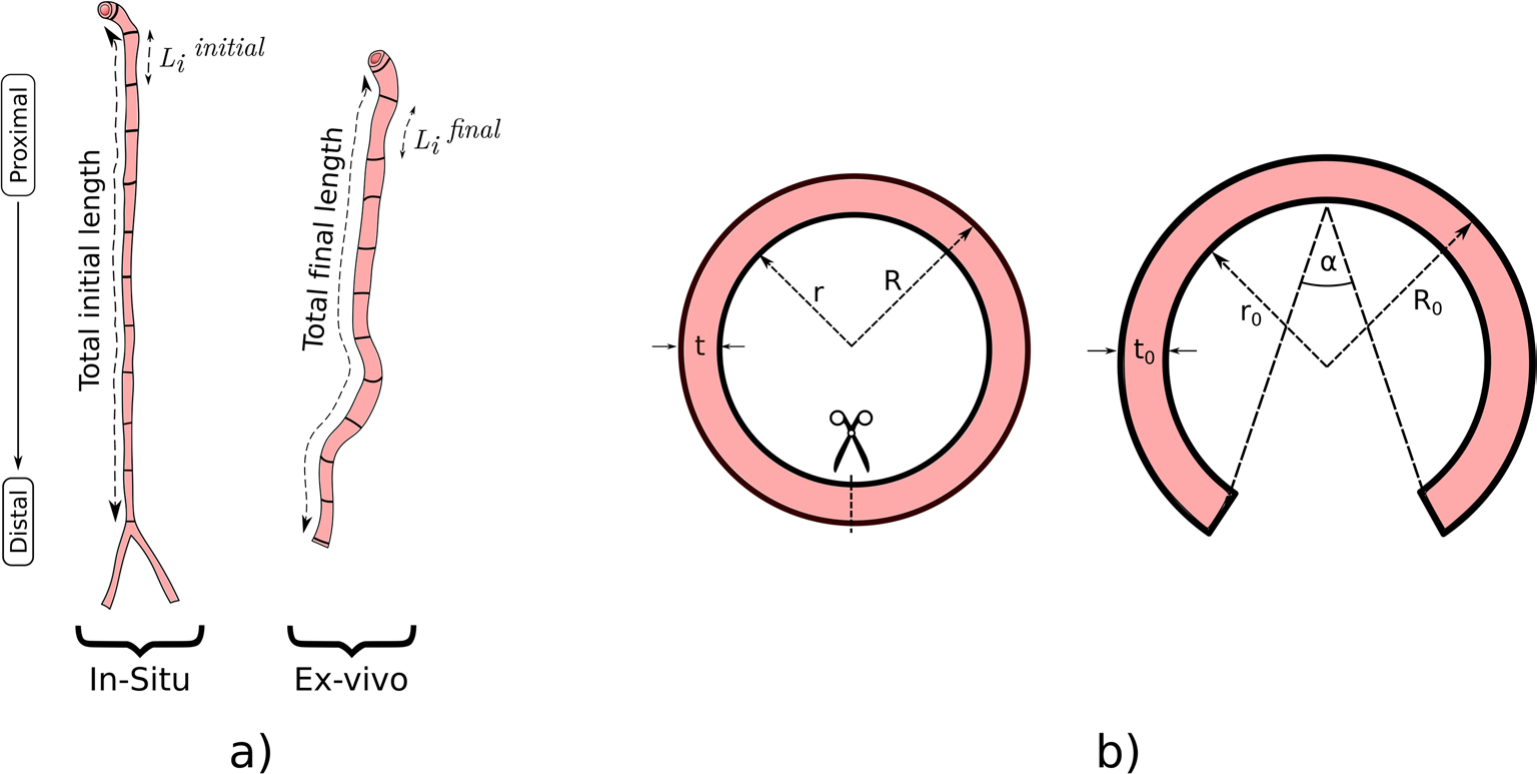
Residual strain tests representation. (**a**) Axial pre-stretch test scheme. (**b**) Ring opening test scheme.

Many studies have presented the impact of the longitudinal stretch in the arterial mechanics, whose effect is essential to reach a homeostatic state in the arteries, maintaining a uniform stress field across the arterial wall in a normal condition^26^, and being related to arterial buckling, tortuosity and kinking conditions^27^. To observe the longitudinal stretch under in-vivo configurations, an axial pre-stretch test was carried out (Fig. 2a)^4,24^. Once euthanasia was performed, the aorta artery length was measured, inside the body of the individual (in-situ state). Then, the artery was marked equidistantly along its entire length using a gel ink pencil, with an approximate separation of 10 mm, with the aim of quantifying the stretch evolution in different zones of the studied artery. After marking, the aorta was photographed and posteriorly removed from the body, always keeping the proximal–distal orientation identified. After 15 min of stabilization in warm Krebs (38 °C), a second image of the new geometrical configuration of the artery was captured (ex-vivo). All images were taken with a reference distance.

For the measurement of the circumferential residual strain, a ring-opening test was performed, according to the scheme observed in Fig. 2b. Two 1 mm-long ring-shaped specimens were extracted from the thoracic aorta region, which was photographed with a bifocal dissecting microscope connected to a digital camera. Then, these ring specimens were cut radially observing a circumferential opening movement. Due to the viscoelastic behavior of the material, a waiting time of 15 min is established for residual strain stabilization, maintaining the tissue inside a Krebs-filled petri dish in an in-house PID dish warmer at 38 °C.After this time, the opened rings were photographed again, in order to quantify the final opening angle α (Fig. 2b), which is the angle formed by the lines that join each end with the midpoint of the perimeter of the artery^28^. A total of three opening angle measurements is performed on each ring specimen image.

#### Uniaxial tensile test

**Figure 3:**
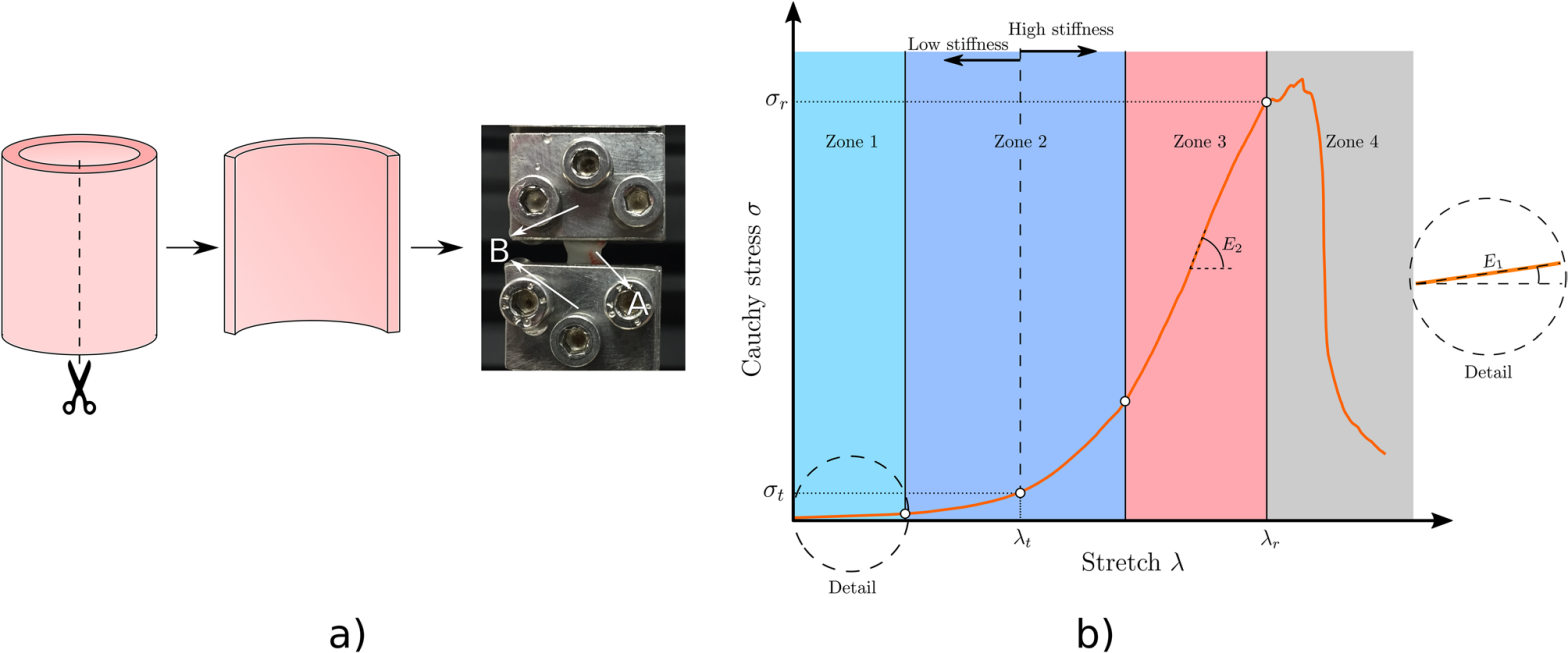
Tensile test representative curve and experimental setup. (**a**) stress-stretch curve of aortic tissue and mechanical parameters. (**b**) experimental setup: A: Longitudinal specimen, B: Steel clamps.

This test is widely used to obtain the stress-stretch relationship of many materials, subjecting a dimensionally measured specimen to a controlled elongation. Longitudinal specimens were tested in the thoracic aorta region, in which a tubular segment is opened over its entire length, with the aid of microsurgical scissors, obtaining a specimen (Fig. 3a), whose initial dimensions, characterized by length ($l_{0}$), width ($w_{0}$) and thickness ($e_{0}$), are appropriately measured by means of image analysis. These tests were performed using an Instron universal testing machine (Instron 3343), with a 10 N load cell and a testing speed of 1.5 mm/min. During the test, the load ($F$) and displacement of the jaws ($\Delta$) were recorded. The stretch ($\lambda$) can be determined by the following equation:

1 $$\lambda = \frac{{l_{0} + \Delta }}{{l_{0} }}$$

Finally, assuming material incompressibility, characteristic of materials with a hyperelastic nature, such as soft tissues^29^, Cauchy stress in the arterial wall is calculated as:

2 $$\sigma = \frac{F}{{w_{0} e_{0} }}\lambda$$

Figure 3b describes the characteristic behavior of an arterial tissue under longitudinal or circumferential loading, presenting a high nonlinear evolution, which has been extensively described elsewhere^30^. These tissues present four well-defined zones across the entire curve: The first one is where the material presents a linear stress-stretch relationship, which can be defined by the slope $E_{1}$. Zone 2 is a transition zone, where the stiffening process takes place. This transition or “elbow” stage is defined by the middle stress and stretch point ($\lambda_{t} ,\sigma_{t}$) between the end of stage 1 and the beginning of the third stage, separating the curve in low and high stiffness regions. Zone 3 is characterized by being almost linear and is the region in which the mechanical damage begins to occur, flattening the previous stage curvature, characterized by a final slope $E_{2}$. Finally, the fourth zone is where the material fails, recording the breaking point of the curve ($\lambda_{r} ,\sigma_{r}$). Additionally, strain energy ($E_{t}$ and $E_{r}$) is calculated, computing a numerical integration of the stress-stretch dataset, using Simpson’s rule, whose integration limits ends in the transition and failure points, respectively.

#### Ex vivo aorta function

The dissection and mounting of the aorta were carried out as described previously^31,32^. Briefly, aorta were dissected, cut in 2 mm rings, and mounted in a multichamber wire myograph (DMT 620, Danish Myotechnologies). Optimal diameter and basal tension was set to an equivalent 80 mmHg before assessing vascular function^32^. Vasoreactivity was evaluated by performing cumulative concentration response curves (CCRCs) to different vasoactive agents. To assess vasoconstriction responses, CCRCs to potassium (K+) and phenylephrine (PE) were performed. In addition, CCRCs to methacholine (MetCh), as an endothelium-dependent vasodilator, and to sodium nitroprusside (SNP), to evaluate the muscle dependent vasodilation, were performed following preconstriction with 10^–6^ M of PE. Contractile responses were expressed in terms of wall tension (mN/mm) or percentage of potassium maximal contraction (%Kmax). Relaxation responses were expressed as a percentage of the contraction induced by PE (%Rmax). For all curves, sensitivity (EC50 or pD2) and maximal response (Emax, Kmax or Rmax) were calculated^32^.

#### Histology

A thoracic aortic ring was extracted from each animal and immersed-fixed in 4% paraformaldehyde for 24 h at 4 °C. The samples were then embedded in paraffin and cut into 5 μm thickness cross sections on a Leitz rotary microtome. The sections were mounted on microscope slides and stained with Elastic van Gieson (EVG) staining. Images were captured at 100× with a microscope (Olympus BX-41) coupled to a digital camera and computer. The analysis of the microphotographs was performed with Image Pro-Plus 6.2 (Media Cybernetics, Inc., Rockville, MD, USA) and ImageJ software^33^. Briefly, luminal, medial, and adventitial perimeters were measured for the estimation of the internal and external diameters. We further calculate intima-media and adventitia thickness, luminal/wall area ratios. In addition, the media cellular density was determined for each artery^34^.

**Figure 4:**
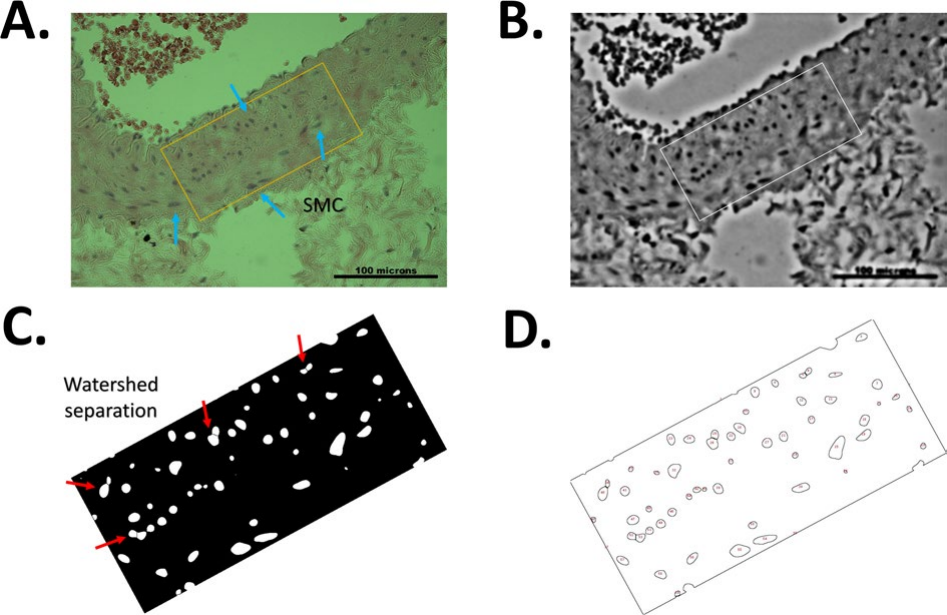
Counting protocol. (**A**) Histology image sample and ROI selection within media layer. (**B**) Gaussian blur and grayscale filter. (**C**) thresholding and watershed separation. (**D**) Particle analyzer result.

In addition, we quantified collagen, elastin, and cell nuclei from the histological photographs through pixel counting method using ImageJ software, described in detail elsewhere^4,25^. From tunica media layer, a region of interest (ROI) was chosen (Fig. 4a), applying a gaussian blur and grayscale filters (Fig. 4b). To isolate the cell nuclei in the ROI, a thresholding filter method was applied in this region (Fig. 4c), and finally, the Particle Analyzer plugin quantifies the cell contours, identifying cell nuclei density within ROI (Fig. 4d). Conversely, elastic fiber quantification in media layer was perform using the color deconvolution plugin for *ImageJ* software, setting previously the representative stain matrix associated to elastin and collagen fibers from staining. From there, elastin and collagen color maps were obtained along with a residual color image, allowing to estimate the fiber content by a pixel area division.

### Statistical analyses

Values are expressed as mean ± S.E.M. All the analyses were carried out with Graphpad Prism 6.01 (GraphPad Software Inc., San Diego, CA, USA). Kolmogorov–Smirnov test was used to check normality. Thereafter, one-way ANOVA, Kruskal–Wallis or two-way ANOVA tests were used accordingly, performing a Tukey post-hoc when multiple comparisons were used. Differences were considered statistically significant when p < 0.05, and finally, all the analyses were performed blind to group assignment.

## Results

### Pre-stretch

**Figure 5:**
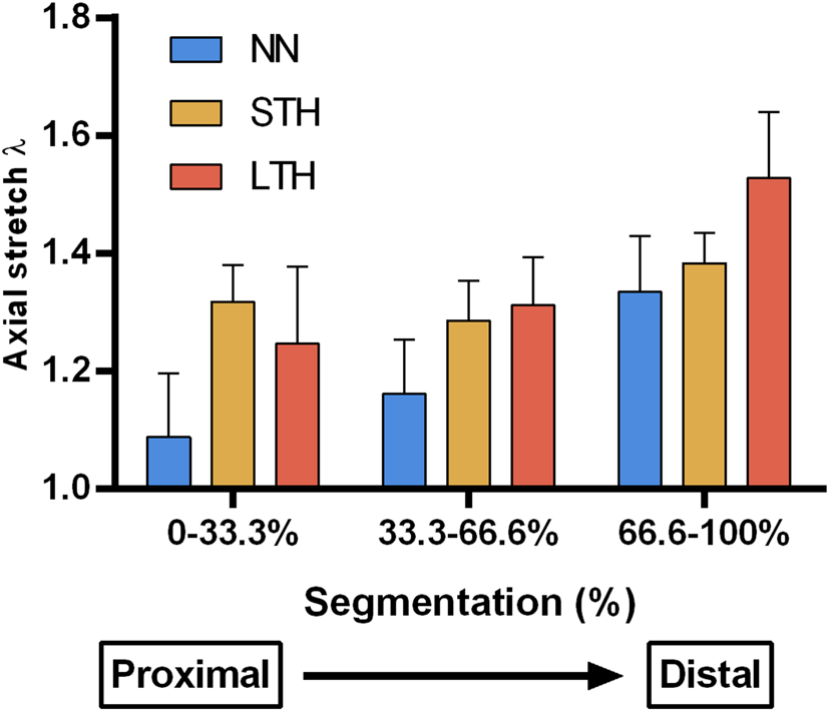
Axial pre-stretch mapping: Each segment represents one region of the thoracic aorta artery, going from the beginning of the artery (proximal) to the thoracic aorta distal end. Values are expressed as mean ± SEM (n = 6 per group).

Figure 5 presents the average pre-stretch values of the analyzed groups. The Two-Way ANOVA analysis, detailed in Table 1, shows several significative differences according to group and region variables. The results show no interaction between segment and group effects (interaction *p* value = 0.12), and the main effects of segmentation and group are analyzed. By means of the segment, there is a significative change of pre-stretch in the second and third segments of the aorta, going from proximal to distal zone. Regarding group assignment, a significative effect of the hypoxia over the pre-stretch values is observed, where both hypoxic groups present differences regarding the control animals.

**Table 1:** Two-Way ANOVA analysis of pre-stretch measurements.

Segment effect	Sig	*p* value	Group effect	Sig	*p* value
0–33.3% versus 33.6–66.6%	ns	–	NN versus STH	*	0.0229
0–33.3% versus 66.6–100%	****	< 0.0001	NN versus LTH	**	0.0061
33.6–66.6% versus 66.6–100%	****	< 0.0001	STH versus LTH	Ns	–

### Ring opening

**Figure 6:**
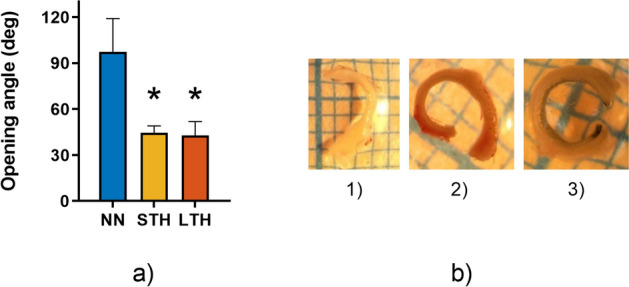
Ring opening test. (**a**) Opening angle measurements in thoracic region of aorta artery. (**b**) image samples of aorta rings (1: NN, 2: STH, 3: LTH). Values are expressed as mean ± SEM (n = 6 per group). Significant difference (*p* ≤ 0.05): *vs NN.

Regarding residual circumferential strains, both hypoxic groups showed a similar marked decrease in the opening angle relative to NN group (Fig. 6). A remarkable aspect regarding opening angle measurements needs to be settled. A high variability (measured as standard deviation per animal) is generally obtained between two consecutive samples of one subject, where the maximum and minimum standard deviation in the entire study was 56.53° and 3.57° respectively. Regarding the reliability of the measurements, the minimum intraclass correlation coefficient (measured as the Cronbach’s alpha coefficient) of the three measurements per sample is as low as 0.904 for the entire dataset. Although the ring opening angle is not capable of describing the residual stress/strain problem, gives a useful mean value of the internal strain state of the vessel. All the raw values can be found in the supplementary material file.

### Mechanical tests

**Figure 7:**
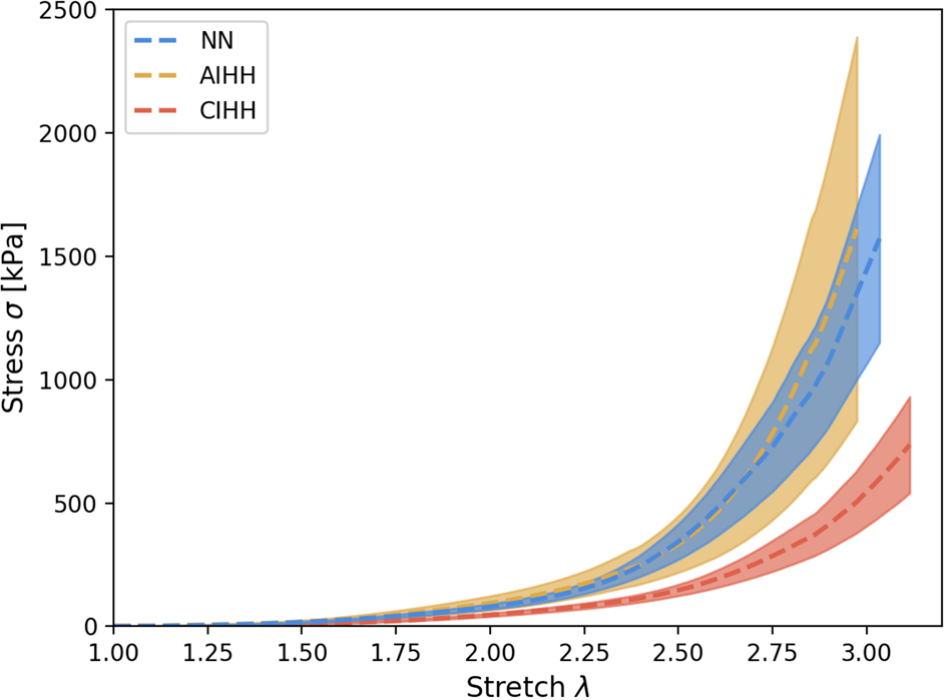
Stress-stretch curves. Stress-stretch mean curves of longitudinal tests on aorta arteries of NN (blue), STH (yellow) and LTH (orange) groups until the first sample rupture. The filled areas represent the error envelope. All values are presented as mean ± S.E.M (n = 6 per group).

Figure 7 shows the average stress-stretch curves of the aorta tensile tests from all groups, until the first sample rupture of each group. The results represent a typical hyperelastic behavior, in which an initial linear stage is followed by a transition stage (stiffening stage), and then by a final linear stage of high stiffness (Fig. 7).

Table 2 shows the average measurements of each group, showing an increased value in both initial and final slopes for the STH group relative to NN group. In contrast, LTH group presented decreased initial and final slopes compared to the NN group. Significant differences were found in the final slope E2 between STH and LTH groups. Finally, at the point of failure of the material, the larger values of rupture stretch, stress, and strain energy correspond to the acute hypoxia group (STH), while the groups control (NN) and long-term hypoxia (LTH) present similar values.

**Table 2:** Mechanical parameters.

		NN	STH	LTH
$E_{1}$	[$kPa$]	13.88 ± 3.12	16.78 ± 3.85	8.01 ± 1.46
$E_{2}$	[$kPa$]	3102 ± 707	4913 ± 633	1711 ± 304†
$\lambda_{t}$		2.61 ± 0.14	3.06 ± 0.06	3.09 ± 0.25
$\sigma_{t}$	[$kPa$]	277.41 ± 64.06	496.95 ± 8.19	508.64 ± 228.91
$\lambda_{r}$		3.80 ± 0.19	4.46 ± 0.18	4.40 ± 0.35
$\sigma_{r}$	[$kPa$]	4272 ± 1168	6595 ± 511	5143 ± 1647
$$E_{t}$$	[$${\raise0.7ex\hbox{${\mu J}$} \!\mathord{\left/ {\vphantom {{\mu J} {mm^{3} }}}\right.\kern-\nulldelimiterspace} \!\lower0.7ex\hbox{${mm^{3} }$}}$$]	124.64 ± 35.18	218.61 ± 17.80	261.14 ± 114.76
$$E_{r}$$	[$${\raise0.7ex\hbox{${\mu J}$} \!\mathord{\left/ {\vphantom {{\mu J} {mm^{3} }}}\right.\kern-\nulldelimiterspace} \!\lower0.7ex\hbox{${mm^{3} }$}}$$]	2149 ± 648	5731 ± 1614	3322 ± 1071

$E_{1}$: initial slope of the stress-stretch curve; $E_{2}$: final slope of the stress-stretch curve; $\lambda_{t}$: stretch at transition point; $\sigma_{t}$: stress at transition point; $\lambda_{r}$: stretch at rupture; $\sigma_{t}$: stress at rupture; $E_{t}$: strain energy at transition stretch; $E_{r}$: strain energy at rupture stretch. Values are expressed as mean ± SEM (n = 6 per group). Significant difference (*p* ≤ 0.05): ^†^versus STH.

### Ex vivo aortic function

**Figure 8:**
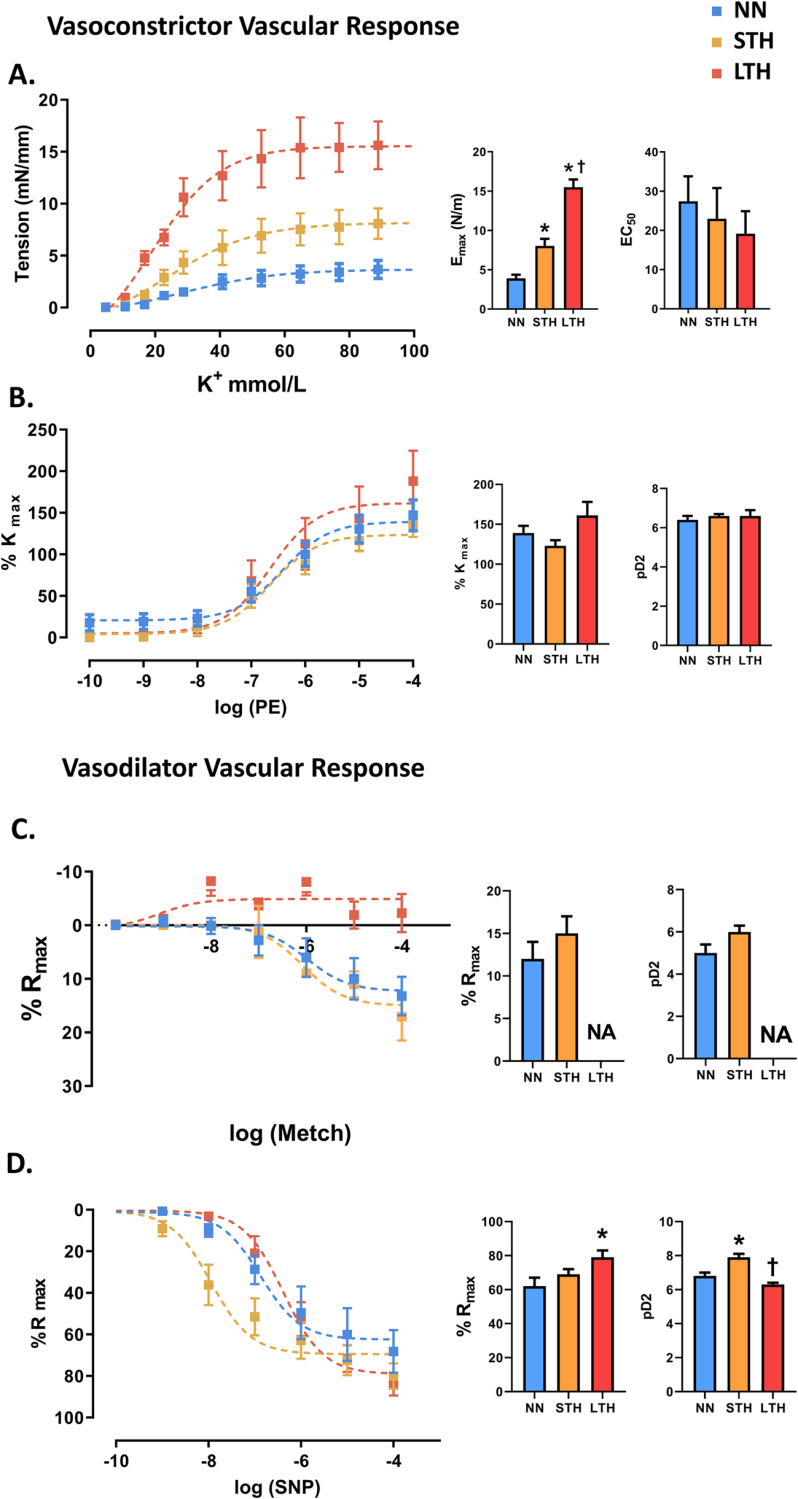
Vasoactive function of aorta artery. Vascular response to potassium (**A**, K+), phenylephrine (**B**, PE), methacholine (**C**, Metch) and sodium nitroprusside (**D**, SNP). Maximal responses (Emax, Kmax or Rmax) and sensitivity (EC50 or pD2) were calculated (inserted histograms). Groups are NN (light blue squares/bars), STH (yellow squares/bars) and LTH (red squares /bars). Values are means ± SEM (n = 6 per group). Significant difference (*p* ≤ 0.05): *versus NN; ^†^versus STH.

The contractile capacity of Aorta in response to potassium increased in both hypoxic groups, being higher in LTH. However, the three experimental groups showed a similar sensitivity (EC50) (Fig. 8A). Further, phenylephrine (PE) induced similar vasoconstriction (%Kmax) and sensitivity (pD2) between groups (Fig. 8B).

We assessed the endothelial-derived vasorelaxation by the MetCh CCR. NN and STH groups showed similar maximal relaxation and sensitivity to MetCh (Fig. 8C). In contrast, endothelial response was totally abolished in LTH. Therefore, pD2 was not assessed in this group (Fig. 8C). In addition, the nitric oxide donor, SNP, revealed an increased maximal relaxation in LTH than STH and NN (Fig. 8D). In addition, the sensitivity to SNP was increased in STH group, when compared to NN and LTH.

### Histology

While STH does not affect aortic internal and external diameters, LTH showed a marked increase of these values relative to normoxic controls. The measurements regarding the intima-media thickness of the samples shows no significative differences between groups. Media/Lumen ratio was decreased only in LTH group, regarding NN, meanwhile STH present no differences with the control group. Finally, the measured cross-sectional area was similar between the three experimental groups (Table 3).

**Table 3:** Histological measurements of the thoracic aorta region.

Group	NN	STH	LTH
**(A)**			
Internal diameter ($\mu m$)	1610 ± 112	1892 ± 56	1962 ± 73*
External diameter ($\mu m$)	2089 ± 96	2367 ± 74	2399 ± 48*
Intima-Media thickness ($\mu m$)	121.4 ± 10.12	128.1 ± 9.77	120.8 ± 10.93
Media / Lumen ratio (−)	1.165 ± 0.01	1.153 ± 0.01	1.123 ± 0.01*
Cross-section area ($\mu m^{2}$)	457,624 ± 35,833	480,155 ± 76,845	485,583 ± 31,759
**(B)**			
Nuclei density ($count/mm^{2}$)	2287 ± 153	2110 ± 90	1985 ± 81
Nuclei size ($\mu m^{2}$)	23.97 ± 1.08	21.59 ± 0.83	21.7 ± 0.51
Nuclei roundness (−)	0.45 ± 0.01	0.47 ± 0.01	0.435 ± 0.01
**(C)**			
% Elastin (−)	25.66 ± 0.69	40.69 ± 3.08*	24.95 ± 1.10^†^
% Collagen (−)	16.53 ± 1.94	21.39 ± 2.92	13.24 ± 1.41

(A) Histomorphometric measurements, (B) Cellular nuclei count and geometric descriptors, (C) Elastic fibers densities within the vascular wall. Values are expressed as mean ± SEM (n = 6 per group). Significant difference (p ≤ 0.05): *versus NN; ^†^versus STH.

In addition, hypoxia exposure does not seem to affect SMC nuclei densities, sizes and roundness, presenting similar values between groups. However, STH group present an increased density of elastin fibers (by 59%) when compared to control (NN) group. Meanwhile, LTH elastin content presents a significative lower value regarding STH (by 39%). No significative differences were found in the elastin content between NN and LTH groups, moreover, collagen fiber measurements were not statistically different in any of the analyzed groups.

## Discussion

This is the first study to determine the effects of different extensions of IHH exposures on the aorta on adults. Our findings showed that IHH induces multidimensional and cycle-dependent alterations in the thoracic aorta, involving functional, biomechanical and structural aspects.

Longitudinal residual strain results showed that there are region and hypoxia condition effects over the descending thoracic aorta region, without interaction between the region and group measurements. Regarding the segment variability of the pre-strain values, the differences are condensed within the second and third region of the aorta vessel. By another hand, the group analysis showed an increased pre-stretch value in both hypoxic groups regarding the control measurements, with no difference between the long- and short-term groups. Similar trends and numerical value ranges can also be observed in Navarrete et al.^4^ and Rivera et al.^24^, whose studies were focused on the effect of chronic permanent hypoxia in neonates. Interestingly, our studies were performed in relatively old rats, confirming a stable tissue response to the performed biomechanical tests. In all zones, the mean axial stretch λ value in LTH group is always higher with respect to the NN group, which suggests a potential change in longitudinal residual strain along the aorta. However, significant differences are observed only in specific zones and therefore it is not possible to state conclusive assessments. On the other hand, circumferential residual strain, quantified by ring opening angle, in presence of intermittent hypoxia condition, shows significant effects in thoracic aorta artery by comparing normoxic and intermittent hypoxia conditions.

The main contribution of residual circumferential and longitudinal strains has been extensively studied in the past decades^35–37^, attributing this behavior to a stress homogenizer mechanism, whose aim is to maintain a homeostatic state in the vessel wall, constituting an adaptation response to injuries, diseases, and external loads. Zhang et al.^26^ investigated the effect of both axial and circumferential strains, as well the effect of surrounding tissues on the vessel wall strains and stresses. Their findings mainly state that the stress field across the arterial wall becomes more uniform as the longitudinal pre-stretch ratio increases. Accordingly, the residual strains measured in this work are relevant in order to accomplish such adaptation processes.

Uniaxial tensile tests performed in this work allow to identify changes in the mechanical properties in longitudinal direction. According to the results, the main impact of hypoxia cycles is observed in $E_{2}$ parameter, interpreted as a tangential Young's module at high strain levels. Significant differences in this parameter are seen among STH and LTH groups, where a drop of $E_{2}$ modulus is experienced when the number of cycles is increased. Regarding the NN group, there are no significant differences with any of the other groups. Based on the evidence reported by different authors^38–40^, which established physiological adaptation in response to hypoxia, along with the evidence of mechanical changes described above, we extend the concept of adaptation in a mechanical sense, when hypoxia cyclical exposures are increased, due to the similarity of the elastic properties among NN and LTH groups, and a previous increment of stiffness in an intermediate stage (STH). Particularly, under acute condition of exposure, PaO_2_ variation determines physiological readjustments related to an increased blood pressure, as reported in humans^41,42^. Therefore, we hypothesized that stiffness evolution from NN and early stages of IH (STH group) is given by a fiber deposition phenomenon related to PaO_2_ variation, followed by an adaptation process towards LTH group. In these conditions, the elastic properties of the material tend to return near the initial homeostatic values given by the normobaric normoxic condition. Histological results support this hypothesis, relating it with alteration in the proportion of load-bearings structural constituent of arterial wall^43^, specifically of the elastic fibers. Our findings indicate a direct relation between elastin content and $E_{2}$ values. Therefore, similarities in passive biomechanical behavior at long-term hypobaric exposures can be explained by an arterial wall remodeling process. It is worth noting that a remodeling effect triggered by the IHH condition is present, according to histomorphometric measurements, since luminal diameter, external diameter, and media/lumen diameter ratio do exhibit significant differences, mainly between the control and the long-term groups. SMC histological measurements does not show any statistical difference between groups. Therefore, changes in residual strain and passive mechanical properties determined in this work are related to modifications in structural composition of the arterial wall. All these changes are triggered by mechanobiological processes at the arterial wall level^44^, and we considered them as adaptive mechanisms to IHH condition. According to the biomechanics performed by Aguilar et al., 2018^15^, stress/strain relationship in femoral artery under 4-cycles of intermittent hypoxia evidence increased high strain’s tangent elastic modulus values (comparable to E2, according to the nomenclature of this work). A similar trend can be observed in the thoracic aorta of the present work, supporting the evidence of an increased elastic modulus at early IH stages, with associated arterial wall stiffness.

Several studies have examined vascular function associated with ageing and chronic hypoxia; however, no studies have assessed the effects of intermittent hypobaric hypoxia in vascular dysfunction of middle-aged animals. Interestingly, hypoxic cycles were associated with an increased contractile capacity of the aorta, shown by the response to cumulative doses of K+. Although there are no changes in cell density in the middle layer that explain the increase in vasoconstriction associated with cell mass, a possible explanation is the differential modulation of hypoxia in the activation of the electric potential of the smooth muscle cell^45^. The best documented hypothesis for hypoxia and vasoconstriction proposes that Ca2+ entry is mediated primarily via voltage-dependent L-type channels due to hypoxia-induced inhibition of voltage-gated K+ channels (KV channels) and consequent depolarization^46^. Although, our ex vivo experiments are performed under controlled normoxic conditions, there must be a mechanism associated with K-induced contraction, rather than the amount of muscle. Therefore, this study shows that the amount of SMC in the aorta is not altered by 4 or 10 cycles of IHH, but it is functionally altered by the cellular pathways that determine an increased contractile capacity. Currently, our experimental design does not allow us to discriminate which mechanisms are related to this response, so further studies shall analyze the involved mechanisms. In contrast, no changes were observed in the vascular response to phenylephrine in-between groups. Finally, although sympathetic innervation is upregulated by hypoxia via receptor α- and β-adrenergic receptors (ARs) as shown in vascular circulation, in which adrenoceptors contribute to hypoxic vasoconstriction by hypoxia^47^, our results did not show significant difference in vasoconstriction by phenylephrine. It has been shown that there is a differential regulation in the expression of adrenoceptors in models of intermittent chronic hypoxia, in particular, the increase of the β2 isoform in relation to the β1 in smooth muscle cells of cardiac tissue^48^. However, in aorta there are no data on the differential regulation by hypoxia in the adrenergic pathway mediated by phenylephrine, and we did not detect any differential responses to PE in between groups. The LTH group did not show an endothelium-dependent vasorelaxation via muscarinic (cholinergic) activation compared to the other experimental groups. Endothelial methacholine activation is a measure of the balance between the activation of vasodilators (e.g., Nitric oxide and Prostacyclin, among others) and vasoconstrictors derived from arachidonic acid (i.e., Thromboxane or Leukotrienes)^49^. Previous studies have shown that hypoxia and senescence decrease nitric oxide synthesis either by uncoupling or decreasing the substrates of endothelial nitric oxide synthase (eNOS), thus decreasing the bioavailability of nitric oxide in the vascular territory such as the aorta^50^. On the other hand, there is an association between the cyclooxygenase 2 isoform (inducible in the vascular territory) and the prostanoid vasoconstrictor pathway such as thromboxane, increasing vasoconstriction in vascular artery^51^. The vasoactive response to MetCh represents the endothelial vasodilator function, which is critical to maintain vascular homeostasis and blood pressure regulation^52^. Therefore, the lack of endothelial-dependent dilatation represent a marked endothelial dysfunction and a potential increased cardiovascular risk^53^ in our model. Interestingly, hypoxia have been shown to increase oxidative stress and be associated with endothelial dysfunction mechanisms^2^.

In contrast to the endothelial dysfunction found, the smooth muscle-dependent vasodilation to sodium nitroprusside (SNP) was increased in the LTH. A possible explanation is that a compensatory effect is generated by less intracellular nitric oxide derived from the endothelium. Therefore, in response to a decreased endothelial vasodilator capacity, SMC may be increasing the expression of the soluble guanylate cyclase protein (GCs), enhancing the response to exogenous nitric oxide given by SNP, in the LTH group.

All of the above describe a complex response of the thoracic aorta exposed to 4 cycles of intermittent hypoxia (STH). Circumferential residual strain, longitudinal pre-stretch, Tangent Young’s module at high strains, histological fiber measurements, and vasoactive function related findings evidence the strong and multidimensional biomechanical responses to IH conditions in early stages of cycling.

Furthermore, the extension to 10 cycles assessed in the LTH group presents a passive biomechanical behavior like the control group, where the elastic modulus at high strains decreases after the short-term exposure. These values are strongly related to a decrease in the elastic fiber measurements, where LTH elastin content is significantly lower than the STH measurements.

Future investigation should be focused on determining the effects of IHH on other arteries and material directions, and undercover the mechanisms behind these effects, in order to better understand how IHH induces functional, mechanical and structural changes. In addition, hemodynamic studies are useful to provide an insight about complementary effects of hypoxia, which can be related to mechanical changes observed in the current work. The development of additional mechanical tests, such as biaxial tensile test, pressurization / inflation test, and relaxation / creep test to determine arterial viscoelastic response can be useful to provide a more extensive overview of the effect of IHH from a biomechanical point of view.

## Conclusion

Intermittent hypobaric hypoxia has shown to trigger multidimensional and cycle-dependent alterations in the thoracic aorta, in both structural and functional ways, triggering changes in the elasticity of the material, and a posterior adaptation and healing, but causing severe vasoactive dysfunction over time. This study gives a better overview of the biomechanical effects of intermittent hypoxia, offering a baseline for future biomechanical analyses and studies. In this sense, according to genetic-based studies, rat model results appropriate to provide insight on human cardiovascular diseases, specifically concerning to their multifaceted complexities^54,55^. A proof of position-dependent longitudinal residual strain changes has been presented in this manuscript; however, the main experiments were performed only on the descending thoracic region of the aorta. This is a limitation that should be fulfilled in future studies, providing a more extensive overview on the entire aorta vessel. Future studies on this model may focus on the biomechanical evolution of the vascular system under IHH cycles and potential pharmacological treatments, to translate this knowledge to the clinical area. Our findings support that IHH alters the aortic function, structure and biomechanical properties and therefore should be considered in preventive health programs for workers exposed to permanent shifts between high and low altitudes.

## Supplementary Information

Supplementary Information.
